# Epidemiology and Ecology of Tularemia in Sweden, 1984–2012

**DOI:** 10.3201/eid2101.140916

**Published:** 2015-01

**Authors:** Amélie Desvars, Maria Furberg, Marika Hjertqvist, Linda Vidman, Anders Sjöstedt, Patrik Rydén, Anders Johansson

**Affiliations:** Umeå University, Umeå, Sweden (A. Desvars, M. Furberg, L. Vidman, A. Sjöstedt, P. Rydén, A. Johansson);; The Public Health Agency of Sweden, Solna, Sweden (M. Hjertqvist)

**Keywords:** tularemia, Francisella spp., Francisella tularensis, Sweden, epidemiology, vector-borne infections, bacteria, zoonoses, surveillance, geographic distribution, ecology

## Abstract

The zoonotic disease tularemia is endemic in large areas of the Northern Hemisphere, but research is lacking on patterns of spatial distribution and connections with ecologic factors. To describe the spatial epidemiology of and identify ecologic risk factors for tularemia incidence in Sweden, we analyzed surveillance data collected over 29 years (1984–2012). A total of 4,830 cases were notified, of which 3,524 met all study inclusion criteria. From the first to the second half of the study period, mean incidence increased 10-fold, from 0.26/100,000 persons during 1984–1998 to 2.47/100,000 persons during 1999–2012 (p<0.001). The incidence of tularemia was higher than expected in the boreal and alpine ecologic regions (p<0.001), and incidence was positively correlated with the presence of lakes and rivers (p<0.001). These results provide a comprehensive epidemiologic description of tularemia in Sweden and illustrate that incidence is higher in locations near lakes and rivers.

## Abstract 2

Geographic distribution of cases was correlated with the locations of lakes and rivers.

Tularemia is a zoonotic disease that causes geographically confined and seasonal outbreaks in many locations in the Northern Hemisphere (*1*–*3*). The highly infectious causative bacterial agent, *Francisella tularensis*, comprises 4 subspecies, but nearly all cases of tularemia are caused by subspecies *tularensis* (type A), the most virulent type, which is found in North America, or subspecies *holarctica* (type B), which is the most widespread species in Europe (*4*). *F. tularensis* can infect humans through bites of arthropods (e.g., mosquitoes, ticks, tabanid flies); inhalation of infectious aerosols; handling of infected animals; or ingestion of contaminated water (*2*,*3*).

Sweden, Finland, and Turkey have reported the highest incidences of tularemia worldwide (*5*). In Sweden and Finland, the most common form of the disease is ulceroglandular tularemia, which is characterized by a skin ulcer at the site of infection and adjacent swollen regional lymph nodes (*6*–*8*). A marked seasonality of tularemia has been reported in Sweden; most cases occur during late summer and early autumn (*8*–*12*). An exception was an outbreak affecting >2,700 persons during late fall and winter in 1966−1967 (*13*). In 2000, large numbers of cases were recorded outside the historically tularemia-endemic northern regions of Sweden, which could indicate a changing geographic pattern of disease (*6*).

*F. tularensis* subspecies *holarctica* naturally infects several mammalian wildlife species, in particular, mice, rabbits, hares, beavers, voles, lemmings, and muskrats (*14*). In Europe, the ticks *Dermacentor reticularis* and *Ixodes ricinus* are vectors for the bacterium (*15*–*17*), although previous research has suggested that mosquito bites are the most frequent route of transmission to humans in Sweden (*6*,*9*,*18*). Furthermore, a relationship between exposure to *F. tularensis* and the presence of lakes and rivers has long been suspected and is repeatedly described in the literature on tularemia (*4*,*19*–*23*). However, the ecologic cycles and environmental reservoirs of tularemia remain largely unknown. Since the 1950s, observed disease patterns have suggested that tularemia foci in nature coincide with a suitable ecosystem at a particular place (*21*,*24*,*25*). According to this theory, disease vectors, hosts, and the pathogen are tied to a particular landscape—that is, an ecologic region—as the environmental determinant that controls disease distribution.

To determine the ecologic factors that contribute to the transmission of *F. tularensis* and the spread of tularemia in Sweden, we examined trends in the epidemiology of tularemia among humans during 1984–2012. We analyzed descriptive epidemiologic data, including the geographic distribution of cases during the study period, and investigated if changes in distribution occurred and if disease was associated with particular ecologic regions and inland water.

## Methods

### Sources of Epidemiologic, Geographic, and Demographic Data

Since 1968, suspected and confirmed tularemia cases have been mandatory reportable diseases as required by the Swedish Communicable Disease and Prevention Act. Data on tularemia cases occurring from September 1984 through December 2012 were collected from the national system for communicable disease surveillance database, maintained by the Public Health Agency of Sweden. Data on patient sex, age, place of residence, suspected location of disease exposure, date of exposure, date of onset of illness, date of diagnosis, and date of notification were retrieved from this database. This study was approved by the Regional Ethical Review Board in Umeå, Sweden (2014-204-31M).

Data on population, land, and water areas were retrieved from Statistics Sweden (*26*). The proportion of land area covered by inland water (defined as lakes and rivers >6 meters wide) was determined by municipality. The areas of the 4 largest lakes of Sweden (Vänern, Vättern, Mälaren, and Hjälmaren) and sea water areas were calculated separately by municipality. Incidences of tularemia, nationwide and by municipality, were calculated on the basis of the number of infections per 100,000 persons and year by using population census data for 1984–2012. The altitude (meters above sea level) at which each tularemia case-patient was exposed to *F. tularensis* and the centroids of the 9,875 postal code areas in Sweden were retrieved by using the 30 arc-second digital elevation model of Europe (*27*) and the extraction toolset of the Spatial Analyst toolbox in ArcGIS version 10.0 (ESRI, Gävle, Sweden). Geographic data were visualized by using ArcMap software in ArcGIS and R software version 2.9.1 (http://www.r-project.org).

### Geographic Coding and Spatial and Temporal Data

For each tularemia case recorded, geographic coordinates (longitude/latitude) were determined for the location of disease exposure and the disease onset date. (For details, Technical Appendix) These data underwent quality coding to enable subsequent analysis with a high level of spatial and temporal certainty.

### Ecologic Regions and Definition of Northern and Southern Sweden

For our study, we used previously defined ecologic regions (e.g., areas defined by the distribution of flora, fauna, geomorphology, climate, and soils) (*28*). The southernmost part of Sweden is nemoral forest (broadleaf forest); north of this region is the boreo-nemoral forest region (mixed deciduous and coniferous forest). Most of the rest of the country is boreal forest (coniferous forests) and is divided into 3 subregions: southern, middle, and northern boreal forest. Alpine tundra is located in northwestern Sweden and is composed of the birch forest, the middle to low alpine forest (grass and shrub heaths, fens), and the high alpine forest (boulder fields). We defined southern Sweden as the region located south of the southern border of the boreal forest region; the area above this boundary was defined as northern Sweden (*29*).

### Local Outbreaks and Outbreak Length

A local outbreak was defined as >4 cases of tularemia during a 30-day period in a municipality. The criterion for a new outbreak was a lag phase of >4 months after the end of the preceding 30-day outbreak period. The mean duration of outbreaks was compared between the northern and southern parts of Sweden by measuring the interquartile ranges of the outbreaks (i.e., the periods during which 50% of cases occurred).

### Statistical Analysis

Categorical data were analyzed by using the χ^2^ test for goodness of fit. Differences between incidence proportions were analyzed by using the 2-tailed 2-proportion z-test with a 95% CI, and nonparametric bootstrapping was used to construct a 95% CI for the relative increase in risk. The Wilcoxon rank-sum test was used to compare differences between groups, and the Spearman rank correlation was used to study the dependencies between variables. The spatial distribution of tularemia cases was compared with a regularly distributed set of points determined by the underlying population of each municipality. A municipality was part of an ecologic region if its geographic centroid was within the borders of the ecologic region. All statistical analyses were conducted in R version 2.9.1.

## Results

### Epidemiologic Characteristics of Tularemia in Sweden

**Figure 1:**
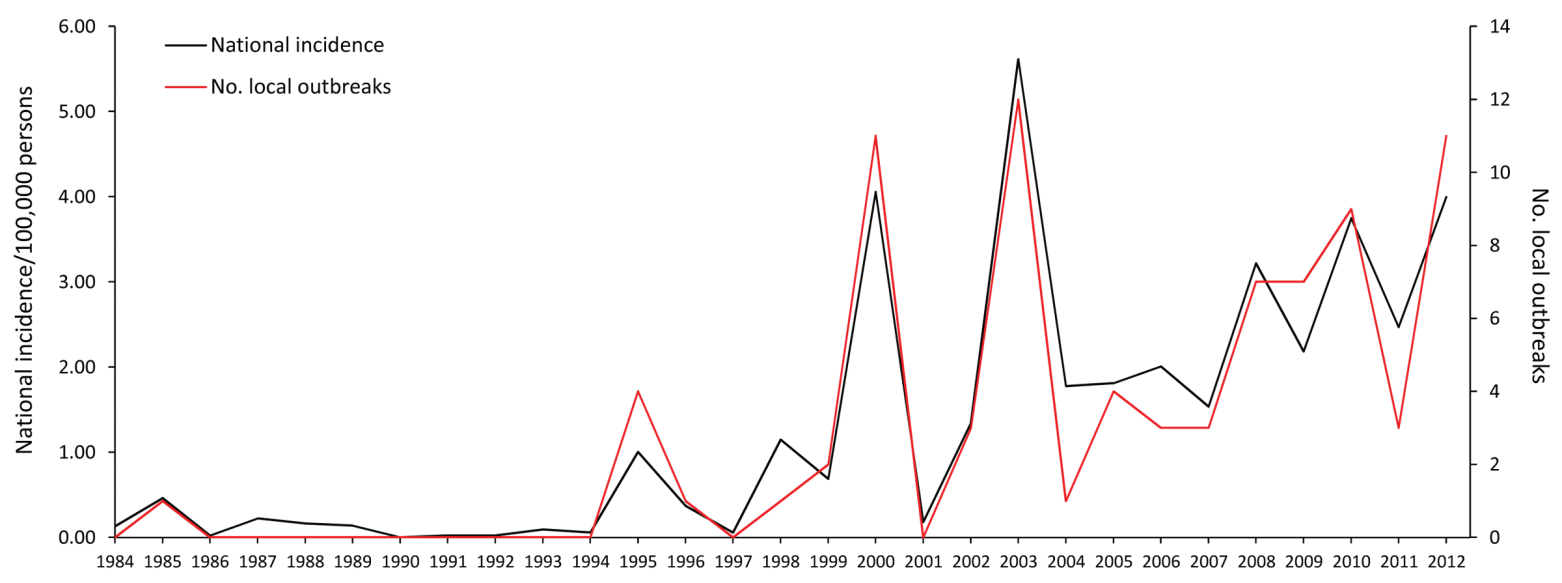
Mean incidence of tularemia (per 100,000 persons) and number of local outbreaks, Sweden, 1984−2012.

During 1984–2012, a total of 4,830 cases of tularemia were notified to the Public Health Agency of Sweden; of these, 4,792 patients were infected in Sweden. The annual mean incidence of tularemia on the basis of these 4,792 patients was 1.86 cases per 100,000 persons (range 0.00−5.62; Figure 1). A total of 2,791 (58.2%) case-patients were men; mean age was 47.6 ± 19.5 years (men, 47.8 ± 19.5; women, 47.2 ± 19.5; range 1−95 years). After applying quality criteria for disease onset date and location of disease exposure, 3,524 of the 4,792 cases were included for the subsequent analyses (the quality of descriptive metadata connected with cases is summarized in Technical Appendix Table 1). All further results, including incidence estimates, were determined on the basis of these 3,524 cases for which high-quality data on disease onset date and location of infection were available.

**Figure 2:**
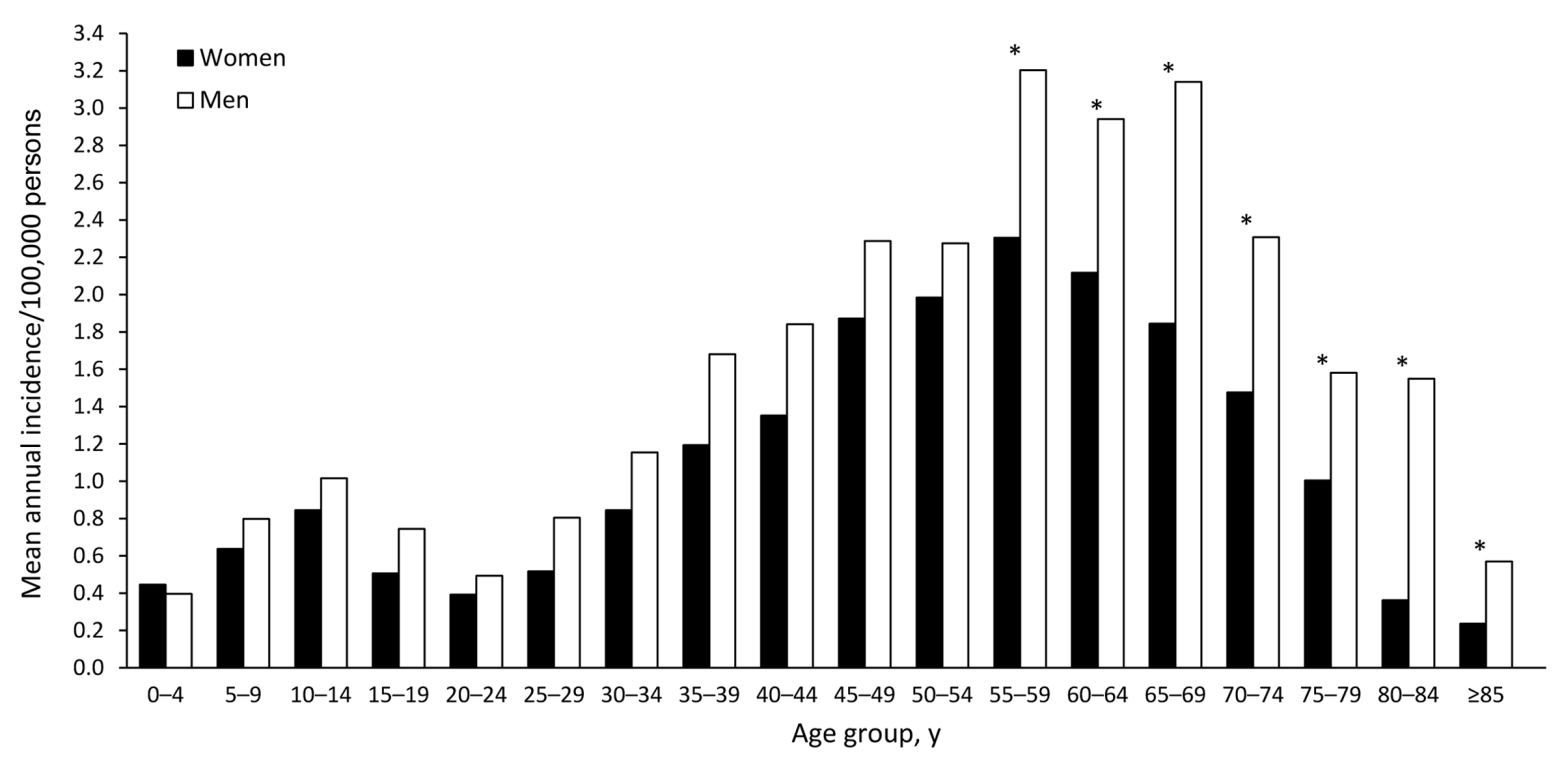
Annual mean incidence of tularemia by age group and sex, Sweden, 1984−2012. Asterisks (*) indicate significant differences by sex.

During the study period, tularemia incidence was distributed widely among age groups, with the highest incidence among those 55–69 years of age (Figure 2). The mean annual incidence by age group showed a distinct bimodal pattern for both sexes; peaks in incidence for age groups 10−14 and 55−59 years were 0.93 and 2.75 cases per 100,000 persons, respectively. The global relative risk of contracting tularemia was 1.39 times higher for men/boys than for women/girls; the corresponding difference in incidence between sexes was 0.44 cases/100,000 persons/year (95% CI 0.35–0.53; p<0.001). By age group, the incidence of tularemia for the study period was significantly higher for men than for women in all age groups >55 years of age (Figure 2). The male:female relative risk for infection was 0.89 for the 0- to 4-year age group but ranged from 1.15 to 4.28 for all other age groups.

### Spatial and Temporal Distribution of Tularemia Cases

**Figure 3:**
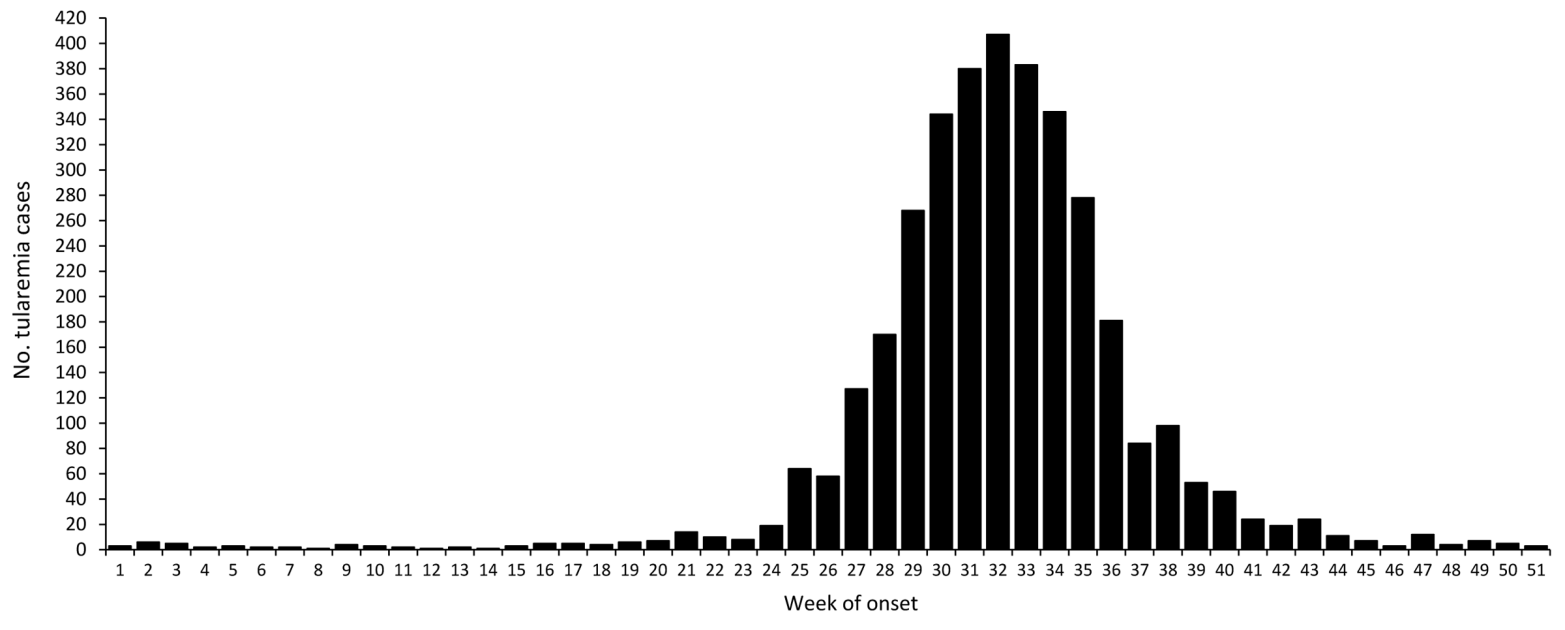
Cumulative number of tularemia cases, by week of onset, Sweden, 1984−2012.

Tularemia reports were highly seasonal. The cumulative number of cases per week for 1984–2012 showed a symmetric pattern with a peak at week 32; approximately two thirds of cases occurred during weeks 29–35 (mid-July to late August; Figure 3). The seasonal outbreak peaks were similarly distributed in the southern region (weeks 30 to 35) and in the northern region (weeks 30 to 34; Wilcoxon rank-sum tests, p>0.05). The mean lengths of outbreaks were also similar between regions (Wilcoxon rank sum test, p>0.05).

**Figure 4:**
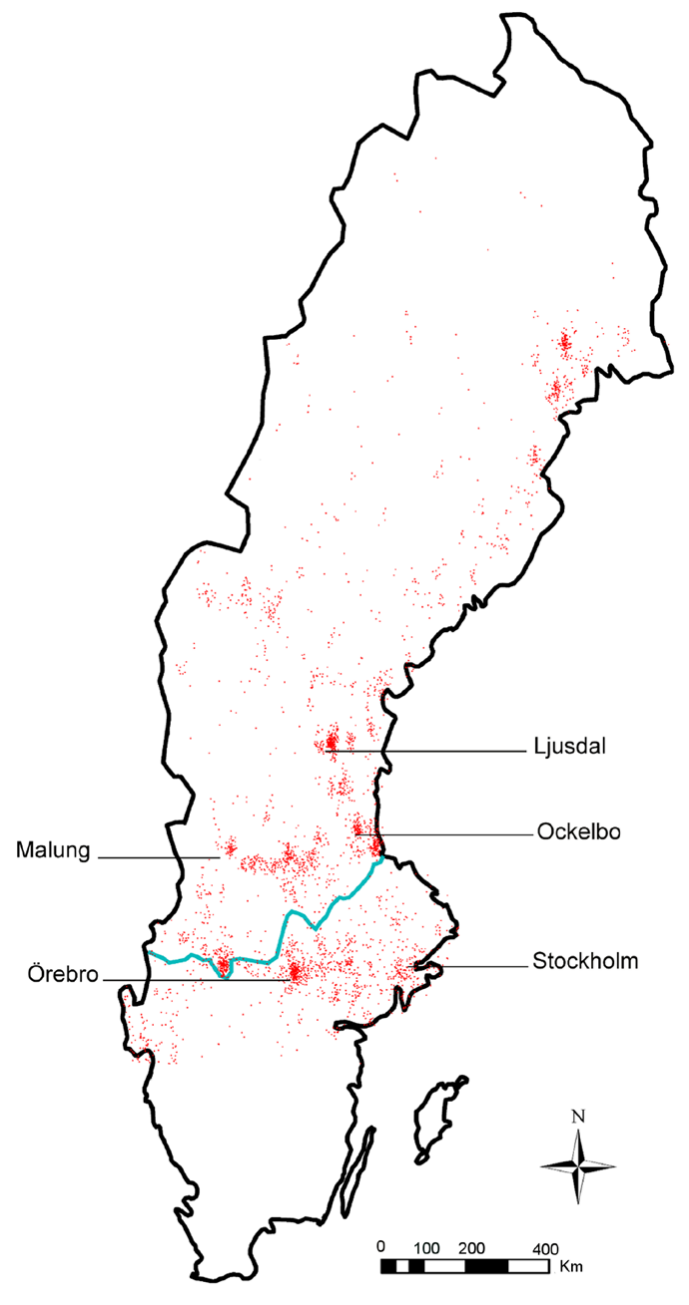
Distribution of 3,524 tularemia cases, Sweden, 1984−2012. Red dots indicate locations of reported cases; blue line indicates border between northern and southern Sweden, as defined by the southern border of the boreal forest. The municipalities with the highest tularemia incidence (Ljusdal, Malung, Ockelbo), and most outbreaks (Örebro) are indicated, as is the capital city of Stockholm.

Tularemia was reported in 189 of 290 municipalities during the study period; some geographic clustering of cases was evident (Figure 4). Most cases were reported from the northern region, where ≈20% of the Swedish population lives, and incidence was significantly higher in the northern region (4.52 cases/100,000 persons/year) than in the southern region (0.56 cases/100,000 persons/year). The 95% CI for estimating the difference in incidence between the northern and southern regions was 3.77–4.14 cases/100,000 persons/year (p<0.001). Denser case aggregates were found in northeast areas of Sweden and in a belt around the southern border of the boreal forest region, which includes the municipalities Ljusdal, Malung, Ockelbo, and Örebro (Figure 4).

The nationwide number of local outbreaks per year varied from 0 to 12 (mean 2.86 ± 3.80) and was largely correlated with the nationwide annual incidence, demonstrating that many local outbreaks occurred simultaneously during outbreak years (Figure 1). The number of outbreaks per municipality during 1984−2012 ranged from 0 to 9 (Technical Appendix Table 2). The highest annual tularemia incidence per municipality was recorded in Ockelbo in 2000 (921 cases/100,000 persons), followed by Malung in 2003 (588/100,000 persons) and Ljusdal in 2008 (429 cases/100,000 persons) and 1998 (402 cases/100,000 persons) (Figure 4).

**Figure 5:**
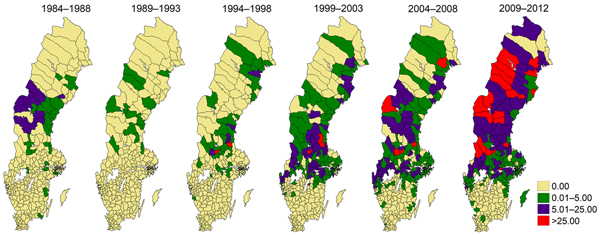
Mean incidence (per 100,000 persons) of tularemia in 189 municipalities by 5-year* intervals, Sweden, 1984−2012. *Most recent interval, 2009–2012, was 4 years.

Analysis showed a significant long-term change in the annual mean incidence of tularemia from the first to the second half of the study period. Incidence was 0.26 cases/100,000 person/year during 1984–1998 but 2.47 cases/100,000 persons/year during 1999–2012 (Table). The 95% CI for estimating the difference in incidence between 1984–1998 and 1999–2012 was 2.16–2.36 cases/100,000 persons/year (p<0.001). An analysis of incidence by municipality showed that, during the first half of the study period, tularemia was mainly reported from municipalities in northern Sweden (Figure 5). However, as the nationwide tularemia incidence increased in the second half of the study period, the disease occurred over a larger geographic area, extending into the southern region. The rate of increase in case reports during the study period was 9.6 times higher in the south than in the north (χ^2^ test, 95% CI 6.37–16.93; p<0.001).

**Table:** Human tularemia notifications, by study period and geographic location, Sweden, 1984–2012*

Study period	No. cases (incidence)		
	Northern Sweden	Southern Sweden	All Sweden
First half, 1984–1998	324 (1.17)	19 (0.02)	343 (0.26)
Second half, 1999–2012	2,034 (8.61)	1,147 (1.14)	3,181 (2.47)
Full study, 1984–2012	2,358 (4.52)	1,166 (0.56)	3,524 (1.37)

*Only cases for which high-quality data on disease onset date and location of disease exposure were available were included. Incidence is given as no. case-patients/100,000 persons/year.

### Tularemia and Ecologic Regions, Altitude, and Inland Water

**Figure 6:**
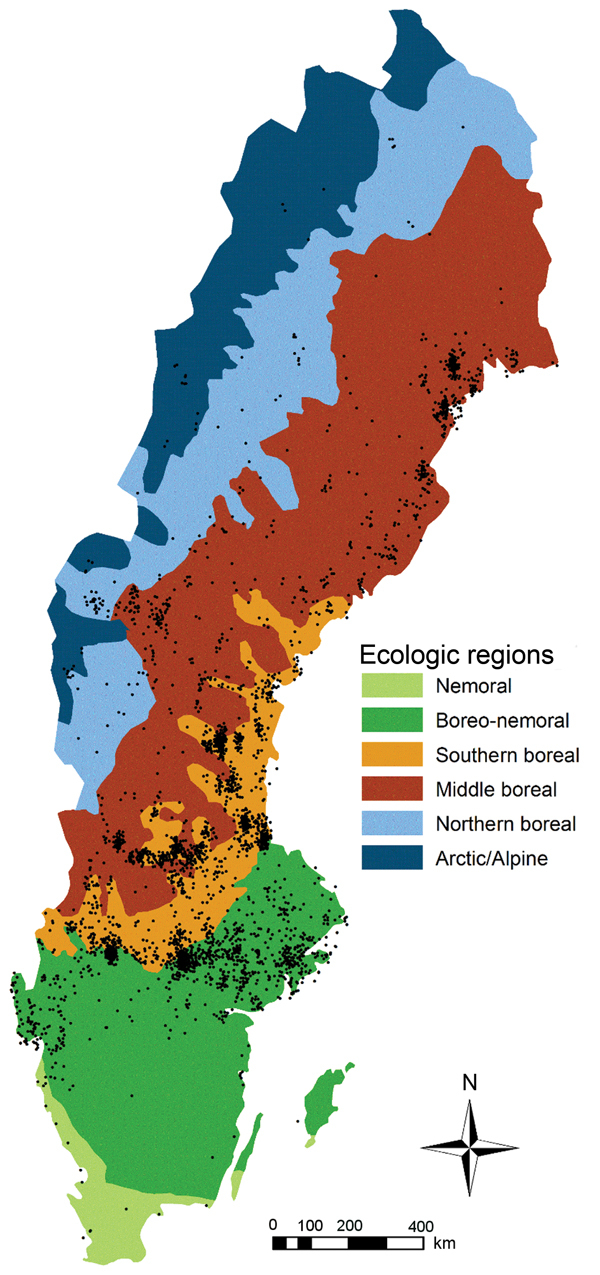
Distribution of tularemia cases by ecologic region, Sweden, 1984−2012. Black dots indicate locations of reported cases. Region designations adopted from (*24*).

The incidence of tularemia was unevenly distributed among the 6 ecologic regions of Sweden (Figure 6). An even disease distribution based on 3,524 cases within the country corresponded with a mean incidence of 1.35 cases/100,000 persons/year. The observed incidences in the nemoral, boreo-nemoral, and combined boreal and alpine regions were 0.02, 0.80, and 4.61 cases/100,000 persons/year, respectively. The 95% CI for measuring the deviation from the mean incidence was −1.42 to −1.23 cases/100,000 persons/year (p<0.001) for the nemoral region; −0.62 to −0.47 cases/100,000 persons/year (p<0.001) for the boreo-nemoral region; and 3.05–3.47 cases/100,000 persons/year (p<0.001) for the combined boreal and alpine region.

**Figure 7:**
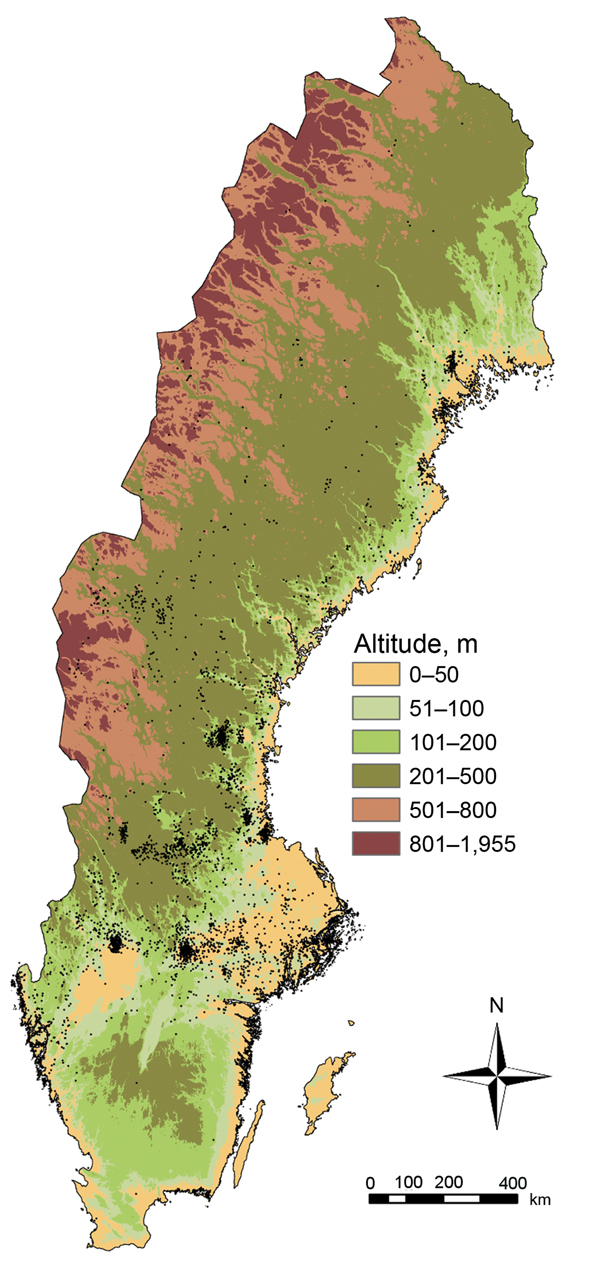
Distribution of tularemia cases by altitude, Sweden, 1984−2012. Black dots indicate locations of reported cases.

Exposure to *F. tularensis* occurred at a median altitude of 59.0 meters (range 0–900 meters); the densest case aggregates were distributed over different altitudes (Figure 7). For municipalities that reported tularemia cases, the mean incidence was 9.27 cases/100,000 persons/year, and a significant positive correlation was seen between the mean altitude of disease exposure and disease incidence (Spearman ρ 0.41, 95% CI 0.28–0.53; p<0.001). The incidence was significantly higher than expected at altitudes >100 meters and lower than expected at altitudes <50 meters (p<0.001). The 95% CIs for measuring the deviation from the mean incidence were 1.08–2.42 cases/100,000 persons/year at altitudes >100 meters and −1.80 to −0.77 cases/100,000 persons/year at altitudes <50 meters.

We found a positive correlation between the mean incidence of tularemia and the proportion of municipality area covered by inland water (Spearman ρ 0.36, 95% CI 0.23–0.47; p<0.001) but a negative correlation between the mean incidence of tularemia and the proportion of the municipality areas covered by sea water (Spearman ρ −0.28, 95% CI −0.40 to –0.15; p<0.05). We found no correlation between the mean incidence per municipality and the proportion of the municipality area covered by the 4 largest lakes in Sweden (Spearman ρ −0.10; p>0.05).

## Discussion

We used 29 years of nationwide notifiable disease surveillance data on tularemia in Sweden to investigate the epidemiologic patterns in relation to time, disease location, and certain ecologic factors. The approach enabled us to identify a marked overrepresentation of cases in the northern part of the country, including annual incidences of 400−900 cases per 100,000 persons in some municipalities, and to identify a marked increase in the overall number of cases reported during the study period. We also observed that the rate of disease increase was higher in the southern part of the country than in the northern part, which suggest that tularemia is becoming more common in the southern regions. We also found statistical support for an association of tularemia with the location of lakes and rivers and with certain ecologic regions of Sweden, a finding notable because the existence of such associations has been postulated for decades but did not have robust support.

We determined that those 40–70 years of age were at greatest risk for tularemia, whereas, for unknown reasons, young adults were at the lowest risk and children and young teenagers were at intermediate risk. A similar age distribution was recently reported from Missouri, USA, which indicates that this type of age distribution is not unique to Sweden (*30*). Age-related differences in disease incidence could mirror differences in behavior; for example, higher-risk outdoor activities such as farming, hunting, and berry-picking may be more common among 40−70-year-olds.

The difference in incidence that we found between the sexes, with tularemia being more common in men than in women, is notable but not unique to this study. Similar findings were reported in several earlier studies (*7*,*13*,*31*). It is unclear if the sex bias results from a difference in exposure to disease or if men are simply more susceptible to tularemia. Our results demonstrate that differences by sex occurred across age groups for all case-patients >5 years of age, a finding that suggests that, among the possible biologic mechanisms involved, sex hormone differences are unlikely to explain the difference in incidence.

Results from our large set of case data corroborated previous reports on the seasonality of tularemia. The disease risk peaks in late summer or early autumn with most cases occurring in early August (*6*,*7*,*9*). In addition, we found that tularemia incidence was almost 10-fold higher in the second half than in the first half of the study period. We cannot rule out the possibility that improved disease awareness accounts for this difference, but because tularemia typically carries distinct symptoms, this is unlikely.

In agreement with previously published data, we observed that the tularemia incidence was highest in the northern part of Sweden and that the disease distribution was uneven, with some municipalities reporting multiple outbreaks (*8*–*10*,*12*). The high risk of contracting tularemia in some municipalities is noteworthy and indicates that tularemia is a public health issue in these locations. Twelve municipalities recorded maximum annual incidences of >100 cases per 100,000 persons and infection transmission occurred almost exclusively during a few summer weeks, findings that offer several possibilities for enhanced preventive measures. Future research efforts can lead to the development of outbreak prediction tools that can help public health authorities make timely decisions on campaigns informing the public on steps to take to prevent infection, such as avoiding exposure to mosquito bites (*18*).

During the second half of the study period, risk for tularemia increased 9.6 times more in southern areas than in northern areas, which indicates that the disease is becoming more common in the southern part of the country. From the available data, we cannot determine whether this shift is occurring because *F. tularensis* is dispersing to new areas or if more infections occurred in the south during the second period because ecologic conditions facilitated increased bacterial multiplication and spread (e.g., through infected arthropod vectors).

A link between tularemia caused by *F. tularensis* subspecies *holarctica* and the location of lakes and rivers was suggested by extensive field work investigating tularemia >50 years ago (*19*–*22*), but we could find little data to prove this association. We found that tularemia incidence at the municipality scale was positively correlated with the proportion of land area covered by inland water (lakes and rivers) but negatively correlated with the proportion of land area covered by sea water. The latter finding can be interpreted as a relatively low incidence of *F. tularensis* infection in municipalities with a long coastline, a finding which contrasts with the distinct association of *F. philomiragia*, *F. novicida*, and some other *Francisella* spp. with sea water environments (*4*).

Interpretation of our finding of underrepresentation of *F. tularensis* exposure at altitudes <50 meters and overrepresentation at altitudes >100 meters is difficult. The most dense geographic case aggregates from 1984–2012 were observed over a range of altitudes, which indicates that intense disease transmission to humans appears to be only marginally restricted by altitude. To provide more informative data, future studies should target areas that experience repeated outbreaks of tularemia and aim for detailed analyses of local disease exposure altitudes and proximity to lakes and rivers. Compilation of chemical and physical characteristics of lake and river waters in areas where disease incidence is high might clarify what kinds of aquatic ecosystems are connected with *F. tularensis*.

We also examined a possible correlation of tularemia with ecologic regions, sometimes referred to as a landscape epidemiology of tularemia (*21*), and found that tularemia cases were overrepresented in the 3 boreal forest regions and the alpine region of Sweden. Combined with the findings described above, these data support a scenario in which the disease is closely related to certain (micro-)environments and ecologic systems (*21*,*24*,*25*).

The strengths of the study include the large sample size and 29-year study period; of the total number of 4,792 cases, 3,524 cases had high-quality descriptive metadata on date of disease onset and location of disease exposure. In 1967, Pollitzer provided a complete overview of the published literature on tularemia in the Soviet Union with greater total case counts, but the raw data used in these older studies are difficult to compare with modern infectious disease surveillance data (*32*). Limitations of our study include the inherent weaknesses of infectious disease surveillance data in general; for example, data on risk factors related to human behavior are lacking, and disease in humans may be underreported because of few clinical symptoms (*33*,*34*). Some inaccurate or imprecise information on disease onset date and location of disease exposure may also have slipped through our filters to ensure strict data quality for case inclusion (e.g., because of patient recall bias).

In conclusion, our findings should stimulate discussion on future possibilities to prevent tularemia. Although this disease does not cause a high number of deaths, the illness can be incapacitating for days, weeks, and sometimes even months. Future studies should focus on the causes of an increased risk for disease in men and in older persons of both sexes. Our findings of a significantly increased risk for contracting tularemia in certain ecologic regions and the positive correlation between disease and inland water may prove useful in future prevention strategies. We believe that knowledge of ecologic region and proximity to water can be used to define areas within which tularemia exposure is more likely. In addition, dynamics introduced by climate change, such as increasing temperature and changing precipitation patterns, can be incorporated in risk assessments (*18*,*35*). Finally, further study is needed to identify the reservoirs of *F. tularensis* in nature and the role of vector abundance. Human activities such as the restoration of wetlands and changes of land use may affect tularemia incidence, but data to influence appropriate risk assessments are lacking.

Technical Appendix Geographic coordinates and quality coding of locations of tularemia disease exposure and disease onset dates, Sweden, 1984–2011.
