# Supplementary material for: Epidemiology and Ecology of Tularemia in Sweden, 1984–2012
Source: Emerg Infect Dis. 2015 Jan;21(1):32–9. doi: 10.3201/eid2101.140916 (PMC4285262; doi:10.3201/eid2101.140916)
Supplement: Technical Appendix — Geographic coordinates and quality coding of locations of tularemia disease exposure and disease onset dates, Sweden, 1984–2011. [file 14-0916-Techapp-s1.pdf]

Article DOI: http://dx.doi.org/10.3201/eid2101.140916

Epidemiology and Ecology of Tularemia in Sweden, 1984–2012

Technical Appendix

Geographic coordinates and quality coding of locations of disease exposure and disease onset dates

Data collected from the national system for communicable disease surveillance at the Public Health Agency of Sweden on the location of disease exposure and the disease onset date were quality coded for each case patient notified. Subsequently, only cases with high-quality data were included for further analyses (Technical Appendix Table 1). The location of disease exposure was selected and a geographical coordinate determined as follows: quality code 1 for a small-sized distinct and unmistakably named disease exposure location, e.g. a small village or the place of residence; quality code 2 for the place of residence if a record in the database stated disease exposure by the municipality name coinciding with the place of residence; quality code 3 for the place of residence if a specific place of disease exposure was not specified; quality code 4 for an unmistakably named disease exposure location if several such potential locations were specified, with the final decision based on an educated guess including knowledge of where other case patients had contracted disease the same year; quality code 5 for a large geographical area (such as a County); and finally quality code 6 for missing data. The disease onset date was determined as follows: quality code A for the disease onset date if this was notified; quality code B for the disease exposure date if the disease onset date was missing; quality code C for the date of disease notification if both the disease onset date and the disease exposure date were missing. We included in this study only case patients with high-quality information on the location of disease exposure (quality codes 1 and 2) and robust information for making an accurate estimation of the disease onset date (quality codes A and B).

Descriptive epidemiological data and geographical coordinates of the local outbreaks are detailed in Technical Appendix Table 2.

Technical Appendix Table 1. Quality coding of time and place data for domestic tularemia notifications 1984–2012 in the Swedish national system for communicable disease surveillance*

Quality code for location of disease exposure	Quality code for disease onset date and corresponding no. case-patients		
	A	B	C
1	**3,326**	**70**	341
2	**142**	**6**	16
3	233	11	64
4	117	4	15
5	164	4	17
6	94	4	164

*Boldface indicates tularemia case-patients that met all study inclusion criteria.

Technical Appendix Table 2. Descriptive data on local outbreaks in Sweden, 1984–2012

Municipalities where local outbreaks occurred	No. outbreaks (years, first outbreak–last outbreak)	Mean incidence*		Cumulative no. cases		Maximum annual incidence (year)	Longitude; latitude in decimal degrees for the centroid of the municipality
		Outbreak years	Nonoutbreak years	Outbreak years	Nonoutbreak years		
Arboga	1 (2010)	105.38	6.34	14	24	105.38 (2010)	59.4000; 15.8333
Arvika	1 (2003)	26.61	2.32	7	17	26.61 (2003)	59.7364; 12.6972
Berg	1 (2010)	163.22	2.88	12	6	163.22 (2010)	62.7725; 14.1699
Boden	4 (2002–2012)	91.28	2.11	101	15	166.68 (2012)	66.0310; 21.1208
Bollnäs	2 (1995–2000)	70.51	3.79	38	27	115.95 (2000)	61.3484; 16.3943
Borlänge	1 (2003)	57.55	2.17	27	29	57.55 (2003)	60.4884; 15.3620
Ekerö	1 (2010)	55.10	3.30	14	22	55.09 (2010)	59.2798; 17.7902
Eskilstuna	2 (2000–2010)	23.77	1.40	44	35	24.92 (2000)	59.3184;16.3833
Falun	1 (2003)	39.99	3.02	22	47	39.99 (2003	60.7169; 15.8428
Flen	1 (2000)	60.37	4.19	10	19	60.37 (2000)	59.0579; 16.5879
Gagnef	5 (1995–2012)	155.86	11.57	79	28	229.72 (2012)	60.4528; 14.8480
Gävle	5 (1985–2012)	28.56	1.44	131	32	57.80 (2003)	60.6749; 17.1413
Hammarö	4 (1999–2009)	86.59	14.81	46	37	172.54 (2010)	59.3115;13.5299
Kalix	1 (2011)	48.22	2.46	8	12	48.22 (2011)	65.9178; 22.8775
Karlstad	4 (2006–2010)	19.01	1.33	64	28	22.42 (2006)	59.3791;13.5008
Katrineholm	1 (2000)	52.52	2.65	17	24	52.52 (2000)	59.0019; 16.4523
Krokom	2 (2008–2011)	58.89	4.92	17	19	62.83 (2008)	63.8201; 14.2943
Köping	1 (2002)	40.57	1.44	10	10	40.57 (2002)	59.5121; 15.9945
Lindesberg	1 (2003)	29.86	2.46	7	16	29.86 (2003)	59.5977; 15.2229
Ljusdal	4 (1995–2008)	276.99	6.97	219	34	428.58 (2008)	61.8308; 16.0818
Ludvika	1 (2003)	73.05	1.52	19	11	73.05 (2003)	60.2051; 14.9393
Luleå	1 (2012)	21.36	0.83	16	17	21.36 (2012)	65.6611; 21.9320
Malung	1 (2003)	588.35	10.47	62	31	588.35 (2003)	60.6864;13.7210
Nordanstig	2 (2003–2005)	95.84	9.36	19	26	100.29 (2003)	62.0359; 17.2048
Nyköping	1 (2000)	18.34	0.98	9	14	18.34 (2000)	58.7528; 17.0085
Ockelbo	3 (1995–2003)	447.47	17.18	83	27	920.99 (2000)	60.8918; 16.7201
Ovanåker	1 (2000)	72.05	4.15	9	14	72.05 (2000)	61.5738; 15.6054
Piteå	3 (1996–2012)	69.87	1.23	86	13	160.67 (2012)	65.3168; 21.4801
Robertsfors	1 (2012)	74.44	3.56	5	7	74.44 (2012)	64.1348; 20.7981
Sandviken	2 (2000–2009)	24.30	0.99	18	10	32.38 (2000)	60.6216; 16.7759
Skellefteå	3 (1999–2012)	16.59	1.17	36	22	20.55 (1999)	64.7048; 20.6358
Strömsund	1 (2010)	49.24	5.16	6	20	49.24 (2010)	63.8522; 15.5678
Sundsvall	2 (2003–2012)	7.87	1.45	15	37	9.31 (2012)	62.3908; 17.3067
Umeå	1 (2008)	7.98	0.44	9	14	7.98 (2008)	63.9565; 20.3265
Vansbro	2 (2003–2008)	190.19	12.99	27	25	279.17 (2003)	60.5099; 14.2253
Ånge	1 (2010)	139.26	4.80	14	15	139.26 (2010)	62.4507; 15.3702
Åre	2 (2008–2012)	62.95	9.58	13	26	68.23 (2008)	63.5856; 12.7115
Älvsbyn	1 (2012)	73.17	2.02	6	5	73.17 (2012)	65.6826; 20.7634
Örebro	9 (2000–2011)	24.71	0.83	286	22	82.35 (2003)	59.2753; 15.2134
Örnsköldsvik	1 (2012)	27.27	1.79	15	28	27.27 (2012)	63.4490; 18.3156
Östersund	1 (2010)	21.88	1.34	13	22	21.88 (2010)	63.2070; 14.8475

*Incidence is given as no. of cases/100,000 persons/year.
